# The Impact of Seasonally Varying Dissolved Organic Matter in Natural Aquatic Environments on the Photodegradation of Pharmaceutical Pollutants

**DOI:** 10.3390/toxics13060450

**Published:** 2025-05-29

**Authors:** Yue Chen, Jingshuang Cui, Fangyuan Cheng, Jiao Qu, Ya-Nan Zhang

**Affiliations:** State Environmental Protection Key Laboratory of Wetland Ecology and Vegetation Restoration, School of Environment, Northeast Normal University, Changchun 130024, China; chenyue@nenu.edu.cn (Y.C.); cuijs@nenu.edu.cn (J.C.); chengfy310@nenu.edu.cn (F.C.); quj100@nenu.edu.cn (J.Q.)

**Keywords:** dissolved organic matter, photodegradation, levofloxacin, sulfamethoxazole, ibuprofen

## Abstract

Photochemical degradation is a major removal pathway for pharmaceutical pollutants in water, and dissolved organic matter (DOM) in water is an important factor affecting this process. This study investigates the differential effects of seasonally-varied dissolved organic matter (DOM) from Songhua River and Liao River on the photodegradation of pharmaceutical pollutants, using levofloxacin (LFX), sulfamethoxazole (SMZ), and ibuprofen (IBP) as target compounds. The results demonstrated that summer and autumn DOM inhibited the photodegradation of LFX and SMZ through light screening and dynamic quenching effects, with inhibition rates of 35.1% and 55.5%, respectively, whereas winter DOM enhanced degradation through photo-oxidation mechanisms. DOM from Songhua River and Liao River significantly promoted the photodegradation of IBP. Quenching experiments showed differences in the contributions of photochemically reactive intermediates (PPRIs) to the photodegradation of different target pollutants, with hydroxyl radicals (•OH) dominating LFX photodegradation (48.79% contribution), excited triplet states of DOM (^3^DOM*) dominating SMZ photodegradation (85.20% contribution), and singlet oxygen (^1^O_2_) dominating IBP photodegradation (79.89% contribution). The photodegradation pathways were elucidated by measuring the photodegradation by-products of the target pollutants: LFX mainly underwent piperazine ring cleavage and oxidative decarboxylation, SMZ underwent isoxazole ring opening and deamination during photodegradation, and IBP underwent photodecarboxylation and oxidation reactions. Under the influence of the DOM from the Songhua River and Liao River, the generation of multiple photodegradation by-products led to an increasing trend in the acute toxicity of target pollutants to luminescent bacteria. This investigation elucidates the dual regulatory mechanisms of natural aquatic DOM on both photo-induced degradation pathways and toxicity evolution dynamics of pharmaceutical contaminants, which is of great significance for understanding the photochemical transformation behavior and risk assessment of pharmaceutical pollutants in aquatic environments.

## 1. Introduction

Global pharmaceutical consumption has surged over recent decades, driven by population growth, advancements in healthcare research and development, expanding global markets, and aging societies in industrialized nations. Currently, about 4000 active pharmaceutical ingredients are marketed worldwide, with annual consumption reaching about 100,000 metric tons [1]. The pervasive release of pharmaceutical pollutants into aquatic environments is now inevitable, with frequent detection in drinking water, sewage, and wastewater treatment plants [2]. Despite trace-level concentrations (ng L^−1^–μg L^−1^) of pharmaceutical contaminants in natural waters, continuous production, use, and discharge have led to their pseudo-persistence, posing risks such as ecosystem disruption and antimicrobial resistance [3].

Pharmaceutical pollutants, as emerging organic contaminants, have been demonstrated to undergo photochemical degradation as their primary transformation pathway in surface waters [4,5]. In aquatic environments, DOM serves as a crucial coexisting component that plays a pivotal role in the photochemical transformation of pharmaceutical pollutants [6]. DOM exhibits dual roles in pharmaceutical pollutants photodegradation. As a photosensitizer, DOM generates photochemically reactive intermediates (PPRIs) that can facilitate the indirect photolysis of pharmaceutical pollutants, particularly for compounds resistant to direct photodegradation [7]. Conversely, DOM may inhibit photodegradation through competitive light absorption and quenching of PPRIs generated during the process [8]. This dual functionality—acting as both promoter and inhibitor—highlights the complex role of DOM in governing the environmental fate of pharmaceutical pollutants in aquatic systems [9,10].

The source of DOM significantly influences its composition and properties, thereby modulating the photochemical transformation of pharmaceutical pollutants [11]. Terrestrial-derived DOM (e.g., from soils) typically generates high concentrations of PPRIs, enhancing the photodegradation of organic contaminants [12]. Marine-derived DOM exhibits lower steady-state PPRI concentrations, resulting in limited photodegradation promotion [13]. DOM from aquaculture-impacted coastal areas demonstrates higher reactivity in facilitating the photodegradation of sulfonamide antibiotics compared to DOM from pristine coastal waters [14]. Notably, DOM sourced from sludge and algal biomass promotes the photodegradation of ritonavir, whereas dust- and soil-derived DOM exhibits inhibitory effects [15]. Furthermore, allochthonous DOM shows stronger inhibition of sulfamethoxazole and trimethoprim photodegradation than autochthonous DOM [16].

Furthermore, in addition to the influence of DOM on the photodegradation efficiency of pollutants, the primary PPRIs generated by DOM from different sources also exhibit distinct effects on pollutant degradation. For instance, Suwannee River fulvic acid primarily affects the photodegradation of amoxicillin through the excited triplet states of DOM (^3^DOM*), whereas peat fulvic acid influences its photodegradation via a combined mechanism involving both the ^3^DOM* and hydroxyl radicals (•OH) [16,17]. In summary, the specific role of DOM in the photodegradation of target pollutants is largely dependent on its origin. Given that the northeastern region of China experiences an annual ice-covered period lasting up to four months, extreme annual temperature variations exceeding 60 °C (https://data.cma.cn/, accessed on 1 March 2025), and fertile black soil, DOM derived from cold regions may differ significantly from that in other areas. Moreover, previous studies on the effects of different DOM types on the photodegradation of organic pollutants have primarily focused on DOM from distinct sources, such as freshwater versus marine DOM. However, no research has yet investigated the seasonal variations in riverine DOM from the same location and their differential impacts on the photodegradation of pharmaceutical contaminants. Prior studies have demonstrated substantial differences in the properties of DOM between dry and wet seasons in the same region, which may further alter its influence on pollutant photodegradation [18]. Since DOM exhibits region-specific characteristics, it is essential to investigate the effects of cold-region riverine DOM and its seasonal variations on the photodegradation of pharmaceutical pollutants. Therefore, relevant studies can help to understand their environmental behavior and migration attenuation mechanism.

This study aims to elucidate the mechanisms by which DOM under varying spatiotemporal conditions influences the photodegradation of pharmaceutical pollutants in aquatic environments. Three high-concentration pharmaceutical contaminants detected in rivers of Jilin Province were selected as target compounds: LFX, SMZ, and IBP [19]. DOM samples were collected from three rivers (Songhua River, Liao River, Yitong River) across different seasons, supplemented with commercial DOM (Suwannee River Natural Organic Matter, SRNOM), to investigate the photodegradation mechanisms of target pollutants in DOM solutions. Quenching experiments were conducted to identify the contributions of PPRIs, and degradation pathways were proposed based on intermediate identification. The ecotoxicological risks of degradation by-products were evaluated. This work systematically examines the differential effects of DOM on pharmaceutical pollutants’ photodegradation behavior, providing fundamental data and a scientific basis for identifying regionally high-risk pollutants. In contrast to previous studies that predominantly focused on single regions or specific types of DOM, this study establishes a spatiotemporal multidimensional framework with distinct geographical characteristics. Earlier research often limited investigations to DOM samples from individual rivers or specific seasons, lacking a comprehensive assessment of DOM differential impacts under varying spatiotemporal conditions. By analyzing DOM samples collected across three rivers and multiple seasons, our work more accurately reflects the spatiotemporal heterogeneity of DOM in natural environments and its influence on the photodegradation of pharmaceutical contaminants. This approach provides robust data support for understanding photochemical processes in complex aquatic systems.

## 2. Materials and Methods

### 2.1. Experimental Reagents and Equipment

LFX, SMZ, and IBP were purchased from Bailiwick Technology (Beijing, China), and Suwanee River I Aquatic Natural Organisms (SRNOM, 1R101N) was purchased from the International Humic Substances Association (St. Paul, MN, USA). Information on the structure of the target drug-like contaminants, among others, is provided in Table S1 in the Supporting Information (SI). The reagents utilized in this research were of analytical reagent grade, and the organic solvents used were of chromatographic purity, with their specifications comprehensively listed in Text S1. All sample solutions were configured using ultrapure water (PW, 18.2 MΩ cm).

### 2.2. Sample Collection and Chemical Analysis

Water samples were collected from the Songhua River, Liao River, and Yitong River (a secondary tributary of the Songhua River) in Jilin Province, China, during February, April, August, and October 2023. Detailed sampling coordinates are provided in Figure S1 and Table S2. DOM was extracted using the method described in Text S2. The basic indexes of the water samples were determined, and the UV-visible absorption spectra and three-dimensional fluorescence spectra of DOM were measured by the method described in Text S3 and Table S3.

### 2.3. Photochemical Experiments

Photodegradation experiments were conducted using an XPA-7 photochemical reactor equipped (Xu Jiang, Nanjing, China), utilizing a 500 W mercury lamp equipped with a 290 nm filter (λ > 290 nm) to mimic the UV-A and UV-B portion of sunlight. The emission spectrum of the mercury lamp is shown in Figure S3. Samples (1.0 mL) were collected at predetermined time intervals for analysis. All photochemical experiments were performed in phosphate-buffered solutions (pH = 7), with dark controls and duplicate experimental sets included for each condition [20]. The photolysis solution contained 20 μM target pollutants and 5 mgC L^−1^ DOM in phosphate buffer (pH = 7). For degradation product analysis, the initial concentration of pharmaceutical pollutants was increased to 25 mg L^−1^ to facilitate the detection of photodegradation products. Specific quenchers were employed to identify reactive species: nitrobenzene (NB, 20 mM) for •OH, sodium azide (NaN_3_, 1 mM) for ^1^O_2_, and sorbic acid (SA, 0.5 mM) for ^3^DOM* [21,22,23].

### 2.4. Toxicity Testing and Toxicity Prediction

The bioluminescent bacterium *Vibrio fischeri* was selected as the test organism for acute toxicity evaluation. The assay was performed as follows: (1) reaction solutions were first adjusted to 3% NaCl osmotic pressure; (2) 100 µL of each test solution was transferred to a 96-well microplate, with 100 µL sterile 3% NaCl solution serving as the control; (3) 50 µL of diluted bacterial suspension was added to each well. The microplate was then placed in a Fluoroskan FL microplate luminometer and mixed by orbital shaking (15 min) prior to luminescence intensity measurement. Toxicity was quantified by the inhibitory effect on bacterial bioluminescence, where greater toxicity corresponds to higher luminescence inhibition. The relative inhibition rate (*I*, %) was calculated as follows:

(1) $$I\;=\frac{L_{B}-L_{S}}{L_{B}}\;\times \;100\%$$

where *L*_B_ represents the luminescence intensity of the blank control and *L*_S_ denotes the luminescence intensity of the test sample.

Evaluation of the toxicity of persistent target pollutants used ECOSAR (Ecological Structure Activity Relationships) to assess the acute toxicity of photodegradation by-products to fish, daphnia, and green algae in ecosystems using the LC_50_ (LC_50_ over 48 h).

### 2.5. Methods of Analysis

Statistical significance between experimental results was determined using the non-parametric Mann–Whitney U test, with all analyses performed using SPSS 25.0 (IBM, Armonk, NY, USA).

Target compound concentrations were quantified by high-performance liquid chromatography (HPLC, 1260 II; Agilent, Beijing, China) equipped with a UV-vis detector and an UltimateTM AQ-C18 column (250 mm × 4.6 mm, 5 μm; Welch Materials, MD, USA). Detailed HPLC operational parameters are provided in Table S4.

Photodegradation products of pharmaceutical pollutants were identified using high-resolution liquid chromatography-mass spectrometry (HRLC-MS; Orbitrap Exploris 120, Thermo Fisher Scientific, Waltham, MA, USA), with instrument parameters described in Text S4. The apparent quantum yields of PPRIs from DOM were calculated as described in Text S5. Indirect photolysis rate constants were determined following the methodology outlined in Text S6.

## 3. Results

### 3.1. Photophysical and Photochemical Properties of Isolated DOM

As shown in Figure S2, the absorbance of seasonal DOM and SRNOM (5 mgC L^−1^) was measured, revealing significantly higher absorbance in the extracted DOM compared to SRNOM, with winter DOM exhibiting the strongest absorbance among all seasonal samples. Combined with UV spectral parameters (Table 1), winter DOM displayed elevated *SUVA*_254_ values, indicating higher aromaticity and a greater abundance of humic-like components with larger molecular weights, which typically exhibit stronger UV absorption [24]. Furthermore, the lower *E*_2_/*E*_3_ ratio of winter DOM confirmed its higher molecular weight characteristics. These macromolecular DOM constituents demonstrated enhanced light absorption extending to longer wavelengths [25]. In contrast, spring DOM underwent more pronounced photobleaching than other seasonal DOM samples and SRNOM, leading to chromophore loss and consequent absorbance reduction.

**Figure 1:**
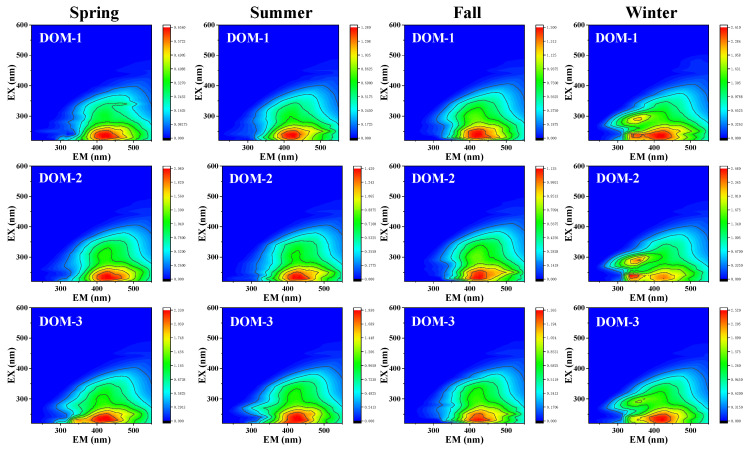
EEM contour maps of DOM for different seasons, color bars are Raman units of fluorescence intensity (R.U).

The three-dimensional fluorescence spectra of DOM in different seasons were analyzed, and the results are shown in Figure 1. It can be observed that the peak Ex/Em wavelengths of DOM in spring, summer, and fall are within the range of 230–260 nm/380–460 nm, primarily indicating fulvic-like substances. Notably, DOM-3 in spring exhibits two peaks, one of which (Ex/Em = 233/250 nm) corresponds to tryptophan-like substances [26,27]. In contrast, winter DOM comprises fulvic-like substances, tryptophan-like substances, and soluble microbial by-products. Due to winter being a period of stagnation, when rivers freeze, and light is reduced, the photochemical degradation of DOM is weakened, and the decomposition rate of microbial metabolites decreases. This leads to the relative accumulation of microbial by-products while further reducing the exogenous input of humic substances [28]. Overall, winter DOM is primarily influenced by local inputs and suppressed degradation due to low temperatures, with endogenous microbial activity playing a dominant role, whereas summer DOM relies more on long-range transport, biological activity, and photochemical degradation.

The differences in DOM light absorption characteristics affected the formation of PPRIs. Simulated sunlight experiments determined the apparent quantum yields (*Φ*_PPRIs_) and steady-state concentrations ([PPRIs]ss) of PPRIs (Table 2). The *Φ*_3DOM*_ values ranged from (0.55–6.03) × 10^−2^, *Φ*_1O2_ from (0.88–10.10) × 10^−2^, and *Φ*_•OH_ from (1.65–10.01) × 10^−5^. The [^3^DOM*]ss, [^1^O_2_]ss, and [•OH]ss concentrations were (0.96–2.29) × 10^−13^ M, (0.38–1.06) × 10^−12^ M, and (0.38–1.49) × 10^−17^ M, respectively. The values of *Φ*_PPRIs_ and [PPRIs]ss from Songliao Basin DOM were higher than those from commercial DOM, with *Φ*_3DOM*_ and [^3^DOM*]ss values in spring, summer, and autumn being one order of magnitude higher. This indicates that the cold-region DOM extracted in this study had higher photochemical activity while showing significant seasonal variations, with winter DOM exhibiting significantly lower quantum yields. The lower *S*_R_ values of winter DOM suggest larger molecular weight and the higher *SUVA*_260_ indicates more hydrophobic components, both contributing to the decreased photochemical activity of winter DOM. Additionally, during winter dry seasons when rivers are ice-covered and water residence time increases, DOM quantum yields decrease, consistent with findings from Grasset et al. [29].

### 3.2. Effect of DOM on Photodegradation of Pharmaceutical Pollutants

**Figure 2:**
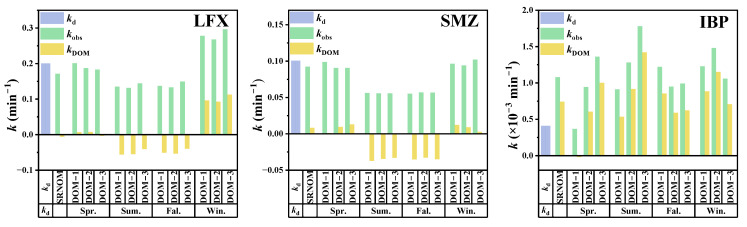
Photolysis rate constants of target pollutants in different DOM solutions (*k*_obs_ represents the apparent first-order degradation rate constant, *k*_d_ denotes the direct photolysis rate constant of the compound in pure water, and *k*_DOM_ refers to the degradation rate constant induced by DOM sensitization).

Dark control experiments showed that the degradation rate of the target pollutants in phosphate buffer solution (pH = 7) with different DOM was less than 5%, indicating that the other degradation pathways could be neglected, and photodegradation was the main degradation pathway under the experimental conditions in this study. The quasi-primary reaction kinetic model fitting showed that the photodegradation rate (*k*) constants of the target pollutants showed significant differences (Figure 2).

For LFX, with a direct photolysis rate of 0.20 ± 0.0060 min^−1^, winter DOM exhibited a photosensitizing effect (*k* = 0.30 ± 0.015 min^−1^), and SRNOM, as well as summer and fall DOM, played an inhibitory role. For SMZ, the values of k constant were 0.10 ± 0.0035 min^−1^, and the DOM samples (spring and winter) did not show significant differences, whereas both summer and fall DOM inhibited its photodegradation, with an average inhibition of 55.50 ± 3.80%. The results indicate that DOM from summer to autumn inhibited the photodegradation of LFX and SMZ, which can be attributed to the DOM in the target river being primarily composed of fulvic acid, which reduced the photodegradation rate of target pollutants through light-screening effects [30]. For IBP, although photolysis occurred at a slower rate ((0.41 ± 0.080) × 10^−3^ min^−1^), it was more obviously affected by DOM, and all DOM samples promoted its degradation, and the maximum degradation rate could reach (0.10 ± 0.050) × 10^−3^ min^−1^.

The effects of DOM on the photodegradation of target pollutants were different in different seasons and sampling sites, and the effects of DOM in cold zones on target pollutants were different from commoditized DOM. This suggests that the observed differences in photodegradation rates are likely attributable to varying production levels of these PPRIs in different DOM systems. To better predict the photodegradation behavior of target pollutants in aquatic environments, we further accounted for DOM light screening effects and calculated the DOM-mediated indirect photolysis rate constants (*k*_DOM_).

The calculated results (Figure 2) reveal negative *k*_DOM_ values for both LFX and SMZ in the presence of summer and autumn DOM, which indicates that DOM not only inhibits direct photodegradation through light screening but also suppresses the process by quenching the excited states of target pollutants [31]. DOM from different seasons exhibited significantly different effects on LFX degradation. Winter DOM showed a clear promoting effect on LFX photodegradation, with *k*_DOM_ values ranging from 0.0924 to 0.1125 min^−1^, while summer and autumn DOM both demonstrated dynamic quenching effects. For SMZ, direct photolysis remained the dominant pathway, with DOM showing relatively weak promoting effects. Specifically, autumn and summer DOM exhibited dynamic quenching effects on SMZ photolysis (*k*_DOM_ < 0). IBP photodegradation was significantly influenced by DOM, with *k*_DOM_ values ranging from (5.88–11.53) × 10^−4^ min^−1^, further confirming that DOM-generated PPRIs promote IBP photodegradation.

### 3.3. Effects of DOM at Different Concentrations on Photodegradation of Pharmaceutical Pollutants

Compared to typical inland rivers, the Songhua River and Liao River exhibit higher total organic carbon (TOC) concentrations in their waters (Table S5). Given this elevated organic carbon concentration characteristic of the Songliao Basin, we established a gradient concentration experimental system (2.5–25 mgC L^−1^) to systematically investigate DOM concentration effects on PPRI generation mechanisms. As shown in Figure S4, while the UV-vis absorption intensity (220–500 nm) of DOM solutions positively correlated with TOC concentration, characteristic absorption parameters showed no concentration dependence, indicating the preservation of conjugated molecular structures in organic matter. According to Table 3, it can be seen that during the photosensitization reaction process, the [PPRIs]ss shows a nonlinear growth trend with the increases in TOC concentration, but the quantum yield shows an inverse decay characteristic, indicating that there is a significant self-shielding effect in the system. When the TOC concentration is higher, the absorbance is higher, the component content is richer, and, therefore, the probability and rate of the self-quenching effect are higher.

**Figure 3:**
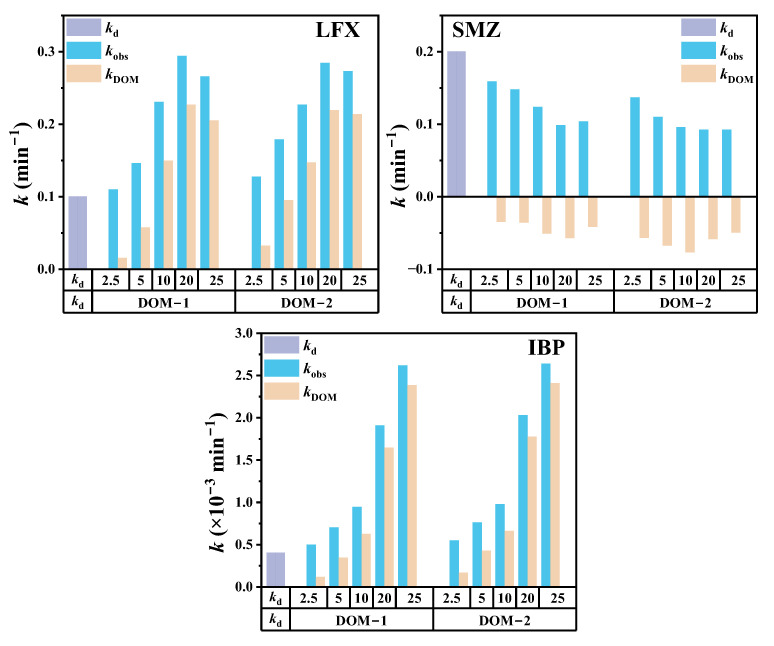
Photolysis rate constants of target pollutants degradation in the presence of different concentrations of DOM. (*k*_obs_ represents the apparent first-order degradation rate constant, *k*_d_ denotes the direct photolysis rate constant of the compound in pure water, and *k*_DOM_ refers to the degradation rate constant induced by DOM sensitization).

The *Φ*_PPRIs_ results indicate that summer DOM exhibited the highest quantum yield, demonstrating strong photochemical reactivity. Therefore, summer DOM-1 and DOM-2 were selected for DOM concentration gradient experiments (2.5–25 mgC L^−1^), which revealed their nonlinear regulatory mechanism on the photodegradation rates of pharmaceuticals (Figure 3). For LFX, which demonstrates rapid direct photolysis and strong photosensitization potential, DOM introduction primarily induced light screening—increasing DOM concentrations elevated optical density, thereby reducing effective photon flux. In contrast, SMZ and IBP, exhibiting weaker inherent photosensitivity, relied predominantly on DOM-mediated PPRIs, resulting in a positive correlation between DOM concentration and degradation rates. However, in LFX systems, at a DOM concentration of 25 mgC L^−1^, the rate of LFX photolysis decreased. This was due to the fact that the high concentration of DOM triggered the self-quenching effect of ^3^DOM*, which inhibited the generation of PPRI and thus slowed down the photodegradation of LFX [32].

### 3.4. Different Roles of PPRIs on Photodegradation of Pharmaceutical Pollutants

**Figure 4:**
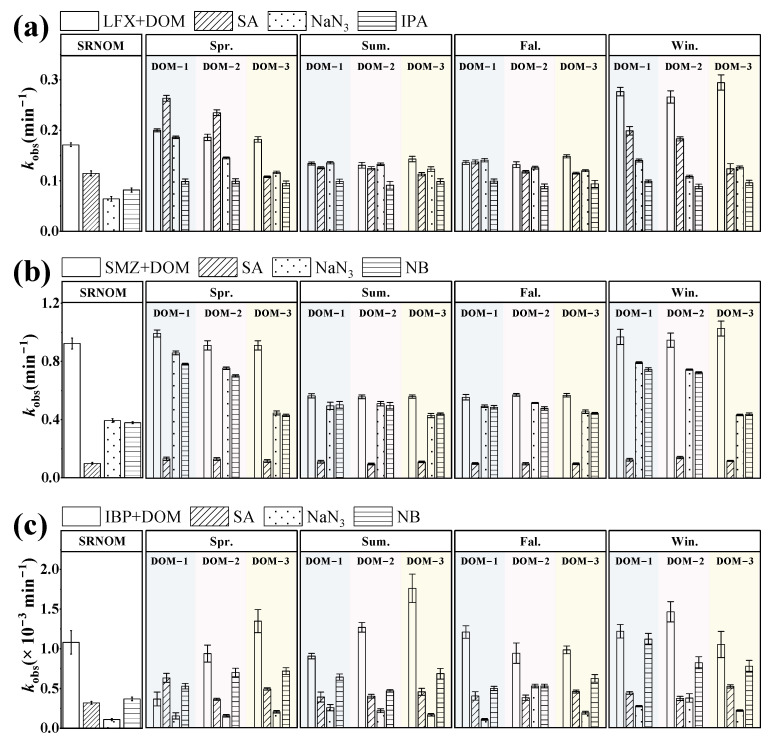
Photolysis rate constants of target pollutants in the presence of DOM with the addition of quencher. (**a**) LFX (**b**) SMZ (**c**) IBP.

Quenching experiments were conducted to clarify the contributions of different PPRIs to the photodegradation of target pollutants, with the results shown in Figure 4. For SMZ, the value of *k* with DOM alone was 0.077 min^−1^. Upon adding quenchers, *k* values decreased significantly to 0.011 min^−1^ (SA), 0.056 min^−1^ (NaN_3_), and 0.054 min^−1^ (NB), indicating ^3^DOM* played the dominant role (85.18% contribution). Notably, commercial DOM generated greater impacts from ^1^O_2_ and •OH compared to Songliao Basin DOM. For IBP, the average k was 1.13 × 10^−3^ min^−1^. Quenching reduced k to 0.44 × 10^−3^ min^−1^ (SA), 0.23 × 10^−3^ min^−1^ (NaN_3_), and 0.66 × 10^−3^ min^−1^ (NB), demonstrating ^1^O_2_ as the primary driver (79.89% contribution). While •OH showed minimal effects, its influence was more pronounced with commercial DOM than with Songliao Basin DOM.

In the presence of DOM, LFX exhibited an average degradation rate constant of 0.19 min^−1^. Given the significant interference of NB with LFX photolysis, isopropanol (IPA) was employed as the •OH quencher in the LFX system. Upon addition of the three quenchers, the degradation rates decreased to 0.15 min^−1^ (SA), 0.13 min^−1^ (NaN_3_), and 0.095 min^−1^ (IPA), respectively, revealing •OH as the predominant reactive species with an average contribution of 48.79%. The results indicate differential contributions of distinct PPRIs to the photodegradation of three target pollutants, suggesting that DOM source characteristics and pollutant molecular structures co-regulate DOM-mediated photodegradation processes [33].

### 3.5. Photodegradation Pathways

To further investigate the influence of DOM in different seasons on the photodegradation of target pollutants and elucidate the regulatory mechanisms of DOM on photolytic pathways, we used DOM extracted from the Songhua River to determine the photodegradation products of the target pollutants in various DOM solutions and proposed potential photodegradation pathways. Specific information on the intermediates is given in Table S6.

**Figure 5:**
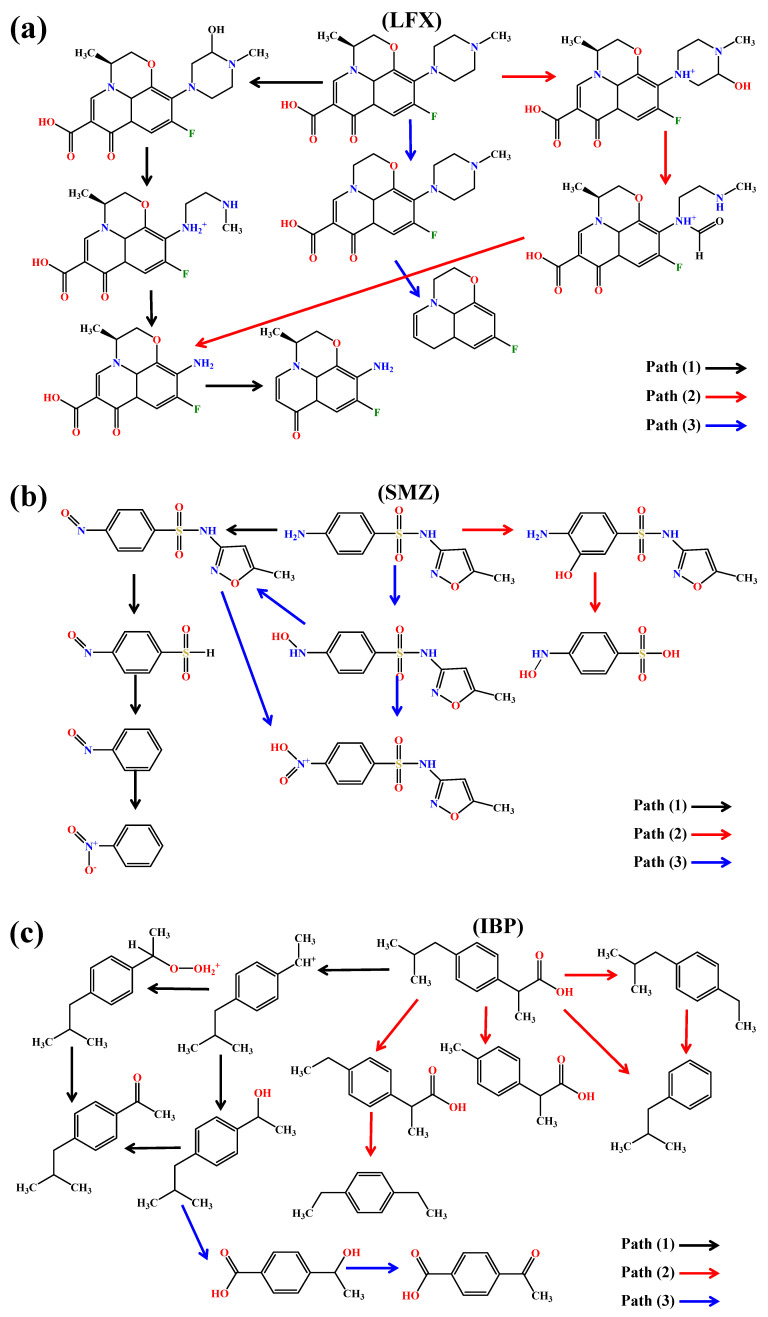
Photochemical degradation pathways of target pollutants in DOM solution ((**a**) LFX, (**b**) SMZ, (**c**) IBP). (DOM concentration: 5 mgC L^−1^; target pollutant concentration: 20 μM).

The primary photodegradation pathways of LFX involved piperazine ring cleavage and oxidative decarboxylation. Distinct degradation mechanisms were observed with seasonal DOM additions (Figure 5a). With spring/summer DOM (Path 1), the N atom in the piperazine substituent served as the reactive site. Initial hydroxylation of the piperazine ring was followed by oxidative cleavage and subsequent decarboxylation. With winter DOM (Path 2), morpholine ring hydroxylation occurred first, followed by piperazine bond cleavage and radical-induced decarboxylation. With autumn DOM (Path 3), •OH attacked the piperazine ring, forming monohydroxy derivatives. Subsequent •OH oxidation caused ring-opening, generating methyl groups that were further oxidized to aldehyde functionalities. The piperazine ring was ultimately removed through continuous •OH oxidation, partially overlapping with Pathway a [34,35].

Under winter DOM conditions (Path 1), SMZ initially underwent hydroxylation followed by two successive oxidation steps (Figure 5b). The DOM-generated ^1^O_2_ preferentially attacked the sulfonyl group (-SO_2_) of SMZ, leading to cleavage of the -SO_2_-NH- bond and formation of sulfonic acid (-SO_3_H). The resulting -SO3H group subsequently hydrolyzed to form new products. With spring DOM (Path 2), the sulfonic acid intermediate (-SO_3_H) underwent further bond cleavage and continued oxidation, generating distinct transformation products. In summer/autumn DOM systems (Path 3), the isoxazole ring of SMZ demonstrated particular susceptibility to oxidative attack, resulting in ring opening accompanied by nitrogen loss. These processes caused significant structural modifications to the SMZ molecule [36,37].

The photodegradation of IBP in DOM solutions generated 4-isobutylacetophenone (4-IBP) as the primary degradation product, with three distinct degradation pathways identified depending on seasonal DOM sources (Figure 5c). Under winter DOM conditions (Path 1), IBP initially underwent photodecarboxylation followed by oxygen addition to the resulting carbon-centered radical, ultimately forming 4-isobutylacetophenone through molecular rearrangement along with the hydroxylated by-product 1-(4-isobutylphenyl)ethanol. Summer DOM induced a different pathway where IBP first decarboxylated and then underwent oxidative attack by highly reactive species on various side chains, generating multiple intermediates that subsequently decarboxylated further (Path 2). In contrast, spring and autumn DOM promoted transformations of alcohol derivatives (Path 3), characterized by hydroxylation of 1-(4-isobutylphenyl)ethanol accompanied by loss of the 2-propanol moiety, yielding more polar transformation products. These differential pathways demonstrate clear seasonal DOM specificity in IBP transformation mechanisms, with winter DOM favoring direct photodecarboxylation and radical recombination and summer DOM causing extensive side-chain oxidation [38,39].

### 3.6. Influence of Dissolved Organic Matter on Contaminant Toxicity

**Figure 6:**
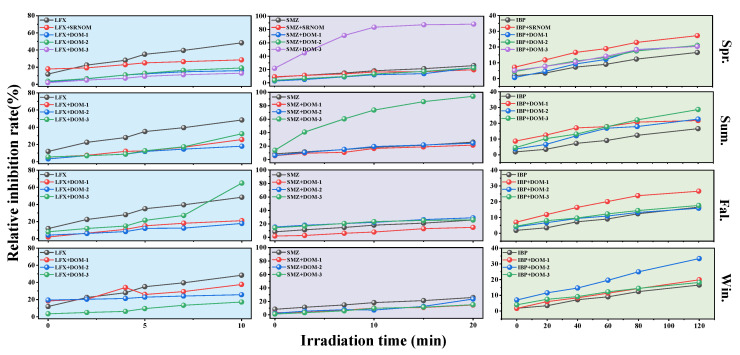
The relative inhibition rates of *Vibrio fischeri* by LFX, SMZ, and IBP as a function of irradiation time in the presence and absence of DOM. (DOM concentration: 5 mgC L^−1^; target pollutant concentration: 20 μM).

An exposure system was established using *Vibrio fischeri* as the test organism to evaluate the toxicity evolution of target pollutants during photodegradation through bioluminescence inhibition assays. As shown in Figure 6, the relative inhibition rates of LFX, SMZ, and IBP on *Vibrio fischeri* gradually increased with prolonged irradiation time during direct photodegradation. However, the addition of DOM in different seasons resulted in distinct trends in the acute toxicity changes of the target pollutants.

The experimental results demonstrated that prior to irradiation, the inhibition rates of different LFX systems on *Vibrio fischeri* ranged from 1.84% to 19.33%. Following 10 min of light exposure, the inhibition rates increased substantially to 12.94–64.93%. The direct photolysis group exhibited a relative inhibition rate of 48.40 ± 3.2% at 10 min, while the addition of sampling site DOM-3 in the fall significantly enhanced the inhibition to 64.93 ± 4.7%. This marked increase may be attributed to the formation of aldehyde intermediates that potentially disrupt bacterial cell membranes [40]. In contrast, other DOM groups showed reduced inhibition rates ranging from 12.94% to 37.62%.

For the SMZ systems, the inhibition rates on *Vibrio fischeri* ranged from 1.27% to 15.44% before irradiation, which increased to 14.56–94.03% after 20 min of light exposure. The direct photolysis group showed an inhibition rate of 25.82 ± 2.1% at 20 min. While most DOM-amended samples showed reduced toxicity inhibition rates (14.56–24.29%), DOM-3 samples from winter and spring demonstrated significantly elevated toxicity with inhibition rates of 94.03% and 87.98%, respectively. This anomalous toxicity may result from specific interactions between sulfonyl groups and key intracellular enzymes of the luminescent bacteria, directly impairing their bioluminescence function and stability [41].

For the IBP systems, the inhibition rates on *Vibrio fischeri* ranged from 0.62% to 8.64% prior to irradiation. Following 2 h of light exposure, the direct photolysis group exhibited an inhibition rate of 16.56 ± 1.8%, while DOM-added groups showed increased inhibition rates ranging from 17.65% to 33.38%. These results demonstrate that while IBP itself displays relatively low acute toxicity to the luminescent bacteria, the ecological risk posed by its degradation products warrants careful consideration. During IBP photodegradation, the formation of 4-isobutylacetophenone—a transformation product with significantly higher toxicity than the parent IBP compound—was identified as a key contributor to the observed toxicity enhancement in DOM-mediated systems.

To further investigate the differences in toxicity among various photodegradation by-products of the target contaminants, their 48-h acute toxicity was predicted, with results presented in Table S7. The analysis revealed that the primary photodegradation by-products of LFX exhibited negligible acute toxicity. In contrast, within the SMZ degradation system, the addition of spring and winter DOM led to a significant increase in toxicity, attributable to the formation of S7, a by-product with notably high acute toxicity. Meanwhile, the major photodegradation by-products of IBP demonstrated relatively strong acute toxicity.

Specifically, while all three compounds showed enhanced toxicity during direct photolysis, DOM-mediated photodegradation systems exhibited variable toxicological profiles, with some DOM treatments mitigating and others exacerbating the toxic effects compared to DOM-free controls. These differential responses highlight the crucial role of DOM characteristics in modulating both degradation pathways and consequent ecotoxicological impacts of pharmaceutical pollutants in aquatic environments.

## 4. Conclusions

This study investigated the effects and mechanisms of DOM from the Songhua River and Liao River on the photochemical degradation of target pharmaceuticals (levofloxacin, sulfamethoxazole, and ibuprofen). The results revealed season-dependent dual inhibition-promotion effects of DOM on LFX and SMX photodegradation: summer/autumn DOM suppressed degradation via light screening and dynamic quenching, while winter DOM enhanced degradation through photo-oxidation. In contrast, DOM consistently promoted IBP photodegradation across seasons. Quenching experiments demonstrated differential contributions of PPRIs: •OH dominated LFX degradation, ^3^DOM* controlled SMX degradation, and ^1^O_2_ mediated IBP degradation. Compound-specific degradation pathways were identified: LFX underwent oxidative decarboxylation and piperazine ring cleavage, SMX experienced isoxazole ring opening with deamination, while IBP degraded via photodecarboxylation followed by oxidation. Notably, acute toxicity to *Vibrio fischeri* increased during photolysis with DOM presence. DOM-mediated photodegradation typically enhanced transformation product toxicity in compound-specific and DOM-specific manners, creating spatially heterogeneous environmental risks in aquatic ecosystems. These toxicity variations, combined with DOM source characteristics and hydrological conditions, ultimately led to significantly different migration and transformation risks across aquatic systems.

In summary, this study systematically investigates the different effects of DOM on the photodegradation behavior of pharmaceutical pollutants and further elucidates the influence of DOM on the photochemical degradation and toxicity evolution of pharmaceutical pollutants in cold regions. The results of this study are of great significance for the in-depth understanding of the photochemical transformation behavior and risk assessment of pharmaceutical pollutants in water in the cold region and provide basic data and a scientific basis for the screening of high-risk pollutants in the region.

## Figures and Tables

**Table 1:** UV-vis spectral parameters and EEM spectral parameters of DOM.

	Site	*SUVA*_254_	*SUVA*_260_	*S*_275–295_	*S*_350–400_	*S*_R_	*E*_2_/*E*_3_
Spr.	DOM-1	2.58	2.35	0.021	0.020	1.32	14.75
	DOM-2	7.69	7.14	0.018	0.019	0.87	7.29
	DOM-3	5.76	5.34	0.020	0.022	1.24	10.15
Sum.	DOM-1	4.01	3.68	0.020	0.016	0.99	7.58
	DOM-2	5.11	4.74	0.018	0.016	0.81	6.16
	DOM-3	6.22	5.62	0.016	0.016	0.85	7.00
Fal.	DOM-1	5.34	4.88	0.016	0.011	0.82	8.13
	DOM-2	5.53	5.11	0.016	0.016	0.77	7.01
	DOM-3	4.51	4.15	0.014	0.022	0.74	7.43
Win.	DOM-1	7.12	6.56	0.017	0.012	0.88	6.81
	DOM-2	9.49	8.84	0.016	0.016	0.76	6.29
	DOM-3	6.43	5.87	0.011	0.005	0.71	7.05
SRNOM		8.64	6.77	0.012	0.017	0.77	5.55

**Table 2:** Quantum yields and steady-state concentrations of ^3^DOM*, ^1^O_2_, and •OH generated from different seasons of DOM.

	Site	*Φ*_3DOM*_ (×10^−2^)	[^3^DOM*]ss (×10^−13^ M)	*Φ*_1O2_ (×10^−2^)	[^1^O_2_]ss (×10^−12^ M)	*Φ*_•OH_ (×10^−5^)	[•OH]ss (×10^−17^ M)
Spr.	DOM-1	6.03	0.96	10.10	0.51	7.07	0.38
	DOM-2	2.03	1.32	4.07	0.83	2.51	0.55
	DOM-3	4.14	1.79	6.31	0.85	1.66	0.12
Sum.	DOM-1	4.94	1.57	6.90	0.69	10.01	1.08
	DOM-2	2.77	1.31	4.71	0.70	9.31	1.49
	DOM-3	4.25	2.29	6.26	1.06	5.09	0.93
Fal.	DOM-1	4.76	1.86	6.13	0.75	7.64	1.01
	DOM-2	2.72	1.26	5.38	0.64	7.90	1.24
	DOM-3	4.11	1.48	7.73	0.83	9.03	1.10
Win.	DOM-1	0.76	1.05	0.88	0.38	2.50	1.17
	DOM-2	0.55	1.02	1.32	0.78	1.65	1.04
	DOM-3	0.87	1.04	2.83	1.06	3.46	1.40
SRNOM		0.41	0.34	1.88	0.31	4.19	5.36

**Table 3:** Quantum yields and steady-state concentrations of ^3^DOM*, ^1^O_2_, and •OH generated from different concentrations of DOM.

Samples	Concentration (mgC L^−1^)	*Φ*_3DOM*_ (×10^−2^)	[^3^DOM*]ss (×10^−13^ M)	*Φ*_1O2_ (×10^−2^)	[^1^O_2_]ss (×10^−12^ M)	*Φ*_•OH_ (×10^−5^)	[•OH]ss (×10^−17^ M)
DOM-1	2.5	7.92	1.49	7.89	1.48	4.13	1.00
	5	4.36	1.64	2.49	1.49	1.76	1.14
	10	2.63	6.94	1.41	3.73	0.91	3.50
	20	1.16	10.62	0.59	5.44	0.44	4.70
	25	0.93	13.14	0.42	5.86	0.27	5.00
DOM-2	2.5	8.38	1.45	6.73	1.17	3.11	0.90
	5	3.70	1.69	1.84	1.34	1.16	1.17
	10	1.59	4.72	1.21	3.61	0.40	2.10
	20	0.60	6.45	0.48	5.18	0.13	4.10
	25	0.50	7.97	0.37	5.98	0.11	4.90
DOM-3	2.5	12.09	1.35	10.25	1.15	3.86	0.80
	5	5.30	1.78	1.80	0.97	1.58	0.85
	10	2.41	5.25	1.45	3.16	0.56	2.50
	20	0.85	6.88	0.60	4.88	0.16	4.20
	25	0.85	10.25	0.45	5.40	0.15	4.50

## Data Availability

The original contributions presented in this study are included in the article. Further inquiries can be directed to the corresponding author.

## Supplementary Materials

The following supporting information can be downloaded at https://www.mdpi.com/article/10.3390/toxics13060450/s1: Text S1: Chemicals; Text S2: Solid phase extraction of river DOM; Text S3: Ultraviolet-visible absorption spectral analysis and fluorescence spectra measurements; Text S4: Test parameters for high-resolution liquid chromatography-mass spectrometry; Text S5: Calculation of formation rates, steady-state concentrations, and apparent quantum yields of PPRIs; Text S6: Calculation of indirect photolysis rate constants; Table S1: Molecular weight and structure values of target pollutant; Table S2: Information of sample points; Table S3: Measurements of optical indices and the corresponding DOM characterization in this study; Table S4: HPLC analysis parameters; Table S5: Water Chemistry of the sample in four seasons; Table S6: The main information on photodegradation products of target pollutants; Table S7: Possible photodegradation by-products and their acute toxicity; Figure S1. Map of sampling sites; Figure S2: UV-vis absorbance of DOM in different seasons and SRNOM; Figure S3: Emission spectrum of the 500 W Hg lamp at λ = 280–400 nm; Figure S4: UV absorption spectra (a) and spectral parameters (b) for different concentrations of DOM. References [42,43,44,45,46,47,48,49] are cited in the supplementary materials.
