# Supplementary material for: The Impact of Seasonally Varying Dissolved Organic Matter in Natural Aquatic Environments on the Photodegradation of Pharmaceutical Pollutants
Source: Toxics. 2025 May 29;13(6):450. doi: 10.3390/toxics13060450 (PMC12197780; doi:10.3390/toxics13060450)
Supplement: Supplementary file 1 [file toxics-13-00450-s001.zip › toxics-3648712-supplementary.pdf]

The impact of seasonally varying dissolved organic matter in natural aquatic environments on the photodegradation of pharmaceutical pollutants

Yue Chen, Jingshuang Cui, Fangyuan Cheng, Jiao Qu, Ya-nan Zhang*

State Environmental Protection Key Laboratory of Wetland Ecology and Vegetation Restoration, School of Environment, Northeast Normal University, Changchun, Jilin 130024, P. R. China

*Correspondence: zhangyn912@nenu.edu.cn

Text S1. Chemicals. 3
Text S2. Solid phase extraction of river DOM. 3
Text S3. Ultraviolet-visible absorption spectral analysis and fluorescence spectra measurements...4
Text S4. Test parameters for high-resolution liquid chromatography-mass spectrometry...4
Text S5. Calculation of formation rates, steady-state concentrations and apparent quantum yields of PPRIs..5
Text S6. Calculation of indirect photolysis rate constants..7
Table S1. Molecular weight and structure values of target pollutant............................9
Table S2. Information of sample points 9
Table S3. Measurements of optical indices and the corresponding DOM characterization in this study...10
Table S4. HPLC analysis parameters (temp: 30 °C) 11
Table S5. Water Chemistry of the sample in four seasons..11
Table S6. The main information on photodegradation products of target pollutants...12
Table S7. Possible photodegradation by-products and their acute toxicity.................14
Figure S1. Map of sampling sites. 17
Figure S2. UV-vis absorbance of DOM in different seasons and SRNOM, [DOM] = 5 mgC L^{-1}. 18
Figure S3. Emission spectrum of the 500 W Hg lamp at λ = 280 - 400 nm. 18
Figure S4. UV absorption spectra (a) and spectral parameters (b) for different concentrations of DOM...18
Supplementary References.. **Error! Bookmark not defined.**

Text S1. Chemicals.

2,4,6-trihydroxybenzophenone (TMP, ≥99%), furfuryl alcohol (FFA, ≥98%), phenol (≥99%), sorbic acid (SA), sodium azide (NaN_3), nitrobenzene (NB), and isopropanol (IPA) were purchased from J&K Scientific Ltd. (Beijing, China). ABTS [2,2'-azino-bis(3-ethylbenzothiazoline-6-sulfonic acid) diammonium salt] was obtained from Shanghai Aladdin Biochemical Technology Co., Ltd. Benzene (98%) was acquired from Sigma-Aldrich Trading Co., Ltd. HPLC-grade methanol and acetonitrile were supplied by Tedia High Purity Solvents Co., Ltd. Sodium dihydrogen phosphate (NaH_2PO_4) and disodium hydrogen phosphate (Na_2HPO_4) were purchased from Tianjin Damao Chemical Reagent Factory (Tianjin, China). PPL solid-phase extraction cartridges (1 g, 6 mL) were procured from Agilent Technologies (USA). Suwannee River I natural organic matter (SRNOM, 1R101N) was obtained from the International Humic Substances Society (IHSS, St. Paul, MN, USA).

Text S2. Solid phase extraction of river DOM.

In this study, water samples were collected using a combined continuous and instantaneous sampling approach, with a total volume of 10 L per sample. All collected samples were protected from light and stored at 4 °C. Upon delivery to the laboratory, the water samples were immediately filtered through 0.45 μm membrane filters to ensure sample stability and analytical accuracy. After DOM extraction, the DOM samples were stored in amber glass centrifuge tubes under light-protected refrigeration and analyzed as soon as possible.

Solid phase extraction (SPE) with Bond Elut PPL small column (1 g/6ml, Agilent) was used to isolate DOM, and the water samples were acidified to pH = 2.0 with formic acid. A total of 10 L water was taken from each river sampling point to extract DOM. SPE cartridges were rinsed with 2 x 5 mL methanol and 2 x 5 mL acidified water. The protocol used was as follows. Start sampling and adjust the flow rate < 5 ml/min. When the sample is running out, wash the ions with 10 - 20 ml water (pH = 2.0 with formic acid) and wash the excess formic acid with 10 - 20 ml ultrapure water. Blow dry the column with nitrogen. Add methanol to the completely dry column and

store the eluate in the sample bottle for later use. Blow dry methanol with nitrogen blower, which can assist hot water bath (warm water) to speed up volatilization.

Text S3. Ultraviolet-visible absorption spectral analysis and fluorescence spectra measurements.

The samples were filtered through a 0.45 μm pore size filter membrane before measurement. The UV-visible absorption spectra of DOM were obtained by a UV-visible spectrophotometer (U-2900, Shimadzu Scientific Instruments, Japan) using Ultrapure water as a blank control. The EEM fluorescence spectra of the samples were taken using a fluorescence spectrometer (F-2700, Hitachi) in 1 cm quartz cuvettes with excitation wavelengths ranging from 220 to 600 nm in 10 nm intervals and emission wavelengths ranging from 220 to 6000 nm in 2 nm intervals.

The EEM data were processed using the Matlab software package, based on the blank sample data minus the effect of scattering and using fluorescence intensity calibrations in Raman units (R.U.) at Ex of 350 nm and Em of 371 - 428 nm, and the data within the fluorescence bands affected by Rayleigh and Raman scattering were removed from the EEMs, using the drEEM 2.0 toolbox in Matlab [42].

Text S4. Test parameters for high-resolution liquid chromatography-mass spectrometry.

The products of photodegradation of pharmaceutical pollutants were determined by high-resolution liquid chromatography-mass spectrometry (HRLC-MS) (Orbitrap Exploris 120, Thermo Fisher). In the high-resolution liquid chromatography-tandem mass spectrometry (HRLC-MS/MS) analysis, a full scan was performed to record the mass-to-charge ratio (m/z) and intensity of all ions in the preset mass range, and the experiment was carried out on an Agilent RRHD Eclipse Plus C^{18} column (2.1 × 50 mm, 1.8 μm), with a resolution of 60,000 for the Orbitrap, and a scanning range of 50-750 for the Orbitrap Exploris 120, Thermo Fisher. The scanning range was 50-750, and the column temperature was maintained at 35 °C. The mass spectrometry conditions for LFX were methanol as mobile phase A and 0.1% formic acid as mobile phase B. The gradient elution program was started from 85% B and held for 1 min, then changed to 75% B within 3 min and held for 10 min, and then changed to 15% B

for 13 min and held for 1 min, and then changed to 85% B for 5 min in the following 0.1 min. The mass spectrometry conditions for SMZ and IBP were methanol as mobile phase A and 0.1% formic acid as mobile phase B. The gradient elution program started with 95% B and was held for 2 min, then it was changed to 80% B in 5 min and held for 3 min, then it was changed to 60% B in 8 min and held for 2 min, then it was changed to 40% B in 10 min, then it was changed to 20% B in 12 min, then it was changed to 15% B in 15 min and held for 3 min to 15% B for 3 min, then quickly changed to 95% B for 2 min.

Text S5. Calculation of formation rates, steady-state concentrations, and apparent quantum yields of PPRIs.

TMP (2,4,6-Trimethylphenol) and ^{3}DOM*. TMP was used as a probe compound to determine the steady-state concentration and quantum yield of 3DOM* [43]: the initial concentration of TMP was 0.1 mM. The steady-state concentration ([3DOM*]ss) and quantum yield (Φ_{3DOM*}) of 3DOM* were expressed by the following equations:

$$\left[{}^3\mathrm{DOM}^*\right]_{\mathrm{SS}} = \frac{R_{^3\mathrm{DOM}^*}}{k_{S,{}^3DOM^*}[\mathrm{S}] + k_{\mathrm{d}}} \quad \text{(S1)}$$

where R_{3DOM*} is the rate of production of ^{3}DOM* in M s^{-1}, which can be calculated by equation (S1); $k_{S,3DOM*}$ is the secondary rate constant for the reaction of the quencher with ^{3}DOM* in M^{-1} s^{-1}; and [S] is the concentration of the quencher in M.

$$R_{^3\mathrm{DOM}^*} = R_{\mathrm{TMP}} \frac{k_{\mathrm{d}} + k_{\mathrm{TMP},{}^3\mathrm{DOM}^*}[\mathrm{TMP}]}{k_{\mathrm{TMP},{}^3\mathrm{DOM}^*}[\mathrm{TMP}]} \quad \text{(S2)}$$

where R_{TMP} is the degradation rate of TMP in the system in M s^{-1}, which can be calculated from the relationship between TMP concentration and light time; $k_{TMP,3DOM*}$ is the secondary rate constant for the reaction of TMP with ^{3}DOM*, 3.0 × 10^9 M^{-1} s^{-1} [44]; and [TMP] is the initial concentration of TMP in M.

$$\Phi_{^3\mathrm{DOM}^*} = \frac{R_{^3\mathrm{DOM}^*}}{\sum_{\lambda} k_{\mathrm{X-a}}(\lambda)[\mathrm{X}]} \quad \text{(S3)}$$

where R_{3DOM*} is the production rate of ^{3}DOM* in M s^{-1}; $k_{X\text{-}a}(\lambda)$ is the characteristic light absorption rate of the photosensitizer X; and [X] is the concentration of the

photosensitizer in M. $k_{X\text{-}a}(\lambda)$ can be calculated by Equation (S3).

FFA (furfuryl alcohol) and $^1O_2$. The steady-state concentration ($[^1O_2]_{SS}$) and quantum yield (Φ_{1O2}) of $^1O_2$ are given by the following equations:

$$\left[{}^{1}\mathrm{O}_2\right]_{\mathrm{SS}} = \frac{R_{{}^{1}\mathrm{O}_2}}{k_{\mathrm{S},{}^{1}O_2}[\mathrm{S}] + k_d} \qquad \text{(S4)}$$

where R_{1O2} is the rate of $^1O_2$ production, which can be calculated by equation (S5); $k_{S,1O2}$ is the secondary rate constant for the reaction of the quencher with $^1O_2$ in M^{-1} s^{-1}; [S] is the concentration of the quencher in M; and k_d is the quenching rate constant for the collision of $^1O_2$ with a water molecule, 2.5×10^5 s^{-1} [45].

$$R_{{}^{1}\mathrm{O}_2} = R_{\mathrm{FFA}} \frac{k_d + k_{\mathrm{FFA},{}^{1}\mathrm{O}_2}[\mathrm{FFA}]}{k_{\mathrm{FFA},{}^{1}\mathrm{O}_2}[\mathrm{FFA}]} \qquad \text{(S5)}$$

where $k_{FFA,1O2}$ is the secondary rate constant for the reaction of FFA with $^1O_2$, 1.0×10^8 M^{-1} s^{-1} [46]; R_{FFA} is the rate of degradation of FFA in M s^{-1} , which can be calculated from the concentration of FFA versus time; and [FFA] is the initial concentration of FFA in M.

$$\Phi_{{}^{1}\mathrm{O}_2} = R_{\mathrm{FFA}} \frac{R_{{}^{1}\mathrm{O}_2}}{\sum_{\lambda} k_{\mathrm{X\text{-}a}}(\lambda)[\mathrm{X}]} \qquad \text{(S6)}$$

where R_{1O2} is the production rate of $^1O_2$ in M s^{-1}; $k_{X\text{-}a}(\lambda)$ is the characteristic light absorption rate of the photosensitizer X; and [X] is the concentration of the photosensitizer in M. $k_{X\text{-}a}(\lambda)$ can be calculated by the following equation:

$$k_{\mathrm{X\text{-}a}}(\lambda) = \frac{I_p \varepsilon_X(\lambda)(1 - 10^{-(\alpha(\lambda)+\varepsilon_{\mathrm{X}}(\lambda)[\mathrm{X}])z})}{(\alpha(\lambda) + \varepsilon_{\mathrm{X}}(\lambda)[\mathrm{X}])z} \qquad \text{(S7)}$$

where I_p is the incident light intensity in Einstein s^{-1} cm^{-2}; $\varepsilon_X(\lambda)$ is the molar absorption coefficient of the photosensitizer in M^{-1} cm^{-1}; $\alpha(\lambda)$ is the unit absorbance of the substrate in cm^{-1}; and z is the optical range in cm.

Benzene and •OH. Benzene was used as a probe compound to determine the rate of •OH production, steady-state concentration, and quantum yield. Benzene can react with •OH to form phenol in a production yield of 0.85, and the steady-state concentration ($[\bullet OH]_{SS}$) and quantum yield ($\Phi_{\bullet OH}$) of -OH are given by the following equations [47]:

$$[\bullet\text{OH}]_{\text{SS}} = \frac{R_{\bullet\text{OH}}}{\sum_i k_{\text{Si},\bullet\text{OH}}[\text{Si}]} \quad \text{(S8)}$$

where $R_{\bullet\text{OH}}$ is the rate of •OH production in mol s^{-1} , which can be calculated by Equation (S9); $k_{\text{Si},\bullet\text{OH}}$ is the secondary rate constant for the reaction of the quencher with •OH in M^{-1} s^{-1} ; and [Si] is the concentration of the quencher in M. The •OH The main quencher was the probe benzene, and the value of $k_{\text{Si},\bullet\text{OH}}$ [Si] was calculated to be 2.34 × 10^7 s^{-1} based on the initial concentration of benzene and the secondary rate constant for the reaction with •OH.

$$\frac{\text{d}[\text{Phenol}]}{\text{d}t} = 0.85 k_{\bullet\text{OH}}[\bullet\text{OH}][\text{Benzene}] = 0.85 R_{\bullet\text{OH}} \quad \text{(S9)}$$

where $k_{\bullet\text{OH}}$ is the secondary rate constant for the reaction of benzene with •OH, 7.8 × 109 M^{-1} s^{-1} [48]; $R_{\bullet\text{OH}}$ is the rate of production of •OH in M s^{-1}; [•OH] and [Benzene] are the system •OH and Benzene concentrations in M, respectively.

$$\Phi_{\bullet\text{OH}} = \frac{R_{\bullet\text{OH}}}{\sum_{\lambda} k_{\text{X-a}}(\lambda)[\text{X}]} \quad \text{(S10)}$$

where is the rate of •OH production in M s^{-1}; $k_{\text{X-a}}(\lambda)$ is the characteristic light absorption rate of photosensitizer X; and [X] is the concentration of photosensitizer in M s^{-1}.

Text S6. Calculation of indirect photolysis rate constants.

The optical shielding factor (S_λ) and the total optical shielding factor ($\sum S_\lambda$) of DOM can be calculated using Equations S11 and S12:

$$S_\lambda = \frac{1\text{-}(10^{-(\alpha_\lambda + \varepsilon_\lambda[\text{T}]l)})}{2.303(\alpha_\lambda + \varepsilon_\lambda[T])\, l} \quad \text{(S11)}$$

$$\sum S_\lambda = \frac{\sum I_\lambda S_\lambda \varepsilon_\lambda}{\sum I_\lambda S_\lambda} \quad \text{(S12)}$$

where α_λ is the light attenuation coefficient of DOM (cm^{-1}); ε_λ is the molar absorption coefficient of the target pollutant (cm^{-1} L·mol^{-1}); and l is the calculated optical path length (cm)..The indirect rate constant (k_{ind}) for DOM-initiated photolysis

can be calculated from Equation S13:

$$k_{\text{ind}} = k_{\text{obs}}(\text{DOM}) - k_{\text{obs}}(\text{non} - \text{DOM})\sum S_{\lambda} \quad \text{(S13)}$$

K_{obs} (DOM) and k_{obs} (non-DOM) are photolysis rate constants observed for the target compounds in the presence and absence of DOM, respectively.

Table S1. Molecular weight and structure values of target pollutant

Compounds	Molecular formula	Molecular structure
Levofloxacin	$C_{18}H_{20}FN_3O_4$	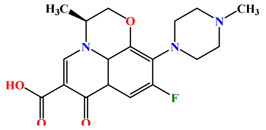
Sulfamethoxazole	$C_{10}H_{11}N_3O_3S$	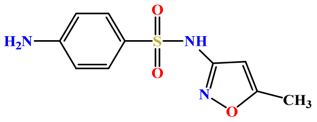
Ibuprofen	$C_{13}H_{18}O_2$	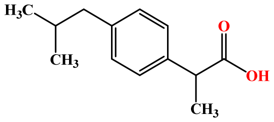

Table S2. Information of sample points

Samples	Longitude (E)	Latitude (N)	Seasons	Temperature (°C)	Climatic
DOM-1	125°44′57″	43°58′12″	Spring	6.1	Sunny
			Summer	23.4	
			Fall	10.6	
			Winter	-2.2	
DOM-2	126°40′49″	43°43′46″	Spring	6.5	
			Summer	23.79	
			Fall	12.7	
			Winter	-0.5	
DOM-3	125°22′43″	42°52′7″	Spring	8.0	
			Summer	25.01	
			Fall	11.28	
			Winter	-1.2	

Table S3. Measurements of optical indices and the corresponding DOM characterization in this study.

Index	Calculation	Description	Reference
SUVA$_{254}$	$SUVA_\lambda = a_\lambda/[DOC]$, where $SUVA_\lambda$ (L mgC^{-1} m^{-1}) is the DOC-normalized absorbance coefficient.	Used to characterize the aromaticity of DOM, the higher the SUVA254, the higher the degree of aromaticity of the organic matter, and the higher the degree of humification	[49]
SUVA$_{250}$	[DOC] is dissolved organic carbon concentration.	SUVA260 is proportional to the content of hydrophobic organic components in DOM	[26]
$S_{275\sim295}$	It is the slope of the absorption spectrum in the 275 ~ 295 nm band.	S275~295 are more sensitive to light radiation and can reflect the degree of DOM photodegradation.S275~295 provide information on the compositional characteristics of DOM, characterizing the molecular weight size and photochemical reaction activity of DOM, etc., and the value is inversely proportional to the molecular weight size.	
E_2/E_3	$E_2/E_3 = A_{(250)}/A_{(365)}$, where E_2/E_3 is the absorbance ratio, $A_{(250)}$ and $A_{(365)}$ were the absorbance at 250 nm and 365 nm, respectively.	Absorbance ratio E_2/E_3 indicates changes in the relative size of DOM molecules: higher E_2/E_3 indicates low molecular weight.	[25]
S_R	$S_R = S_{275-295} / S_{350-400}$, where S_R is the spectral slope ratio and $S_{275-295}$ and $S_{350-400}$ are the spectral slope in the range of 275 -295 nm and 350 - 400nm.	Spectral slope ratio S_R indicates changes in the relative size of DOM molecules: higher S_R indicates lower DOM molecular weight in an increased irradiation.	

Table S4. HPLC analysis parameters(temp: 30℃)

Substances	Mobile phase ratio	Wavelength (nm)	Speeding ($mL\cdot min^{-1}$)
TMP	Acetonitrile：0.1%Phosphoric acid = 45:55	220	1.0
FFA	Acetonitrile：0.1%Phosphoric acid = 15:85	219	1.0
Phenol	Methanol：Water = 45:55	225	1.0
SMZ	Acetonitrile：0.1%Phosphoric acid = 40:60	255	1.0
LFX	Acetonitrile：0.1%Phosphoric acid = 20:80	274	1.0
IBP	Acetonitrile：0.1%Phosphoric acid = 70:30	222	1.0

Table S5. Water chemistry of the sample in four seasons.

	Site	pH	TN ($mg\ L^{-1}$)	TP ($mg\ L^{-1}$)	NH_3-N ($mg\ L^{-1}$)	[TOC] ($mgC\ L^{-1}$)
Spring	DOM-1	8.47	8.61	0.5	0.21	5.90
	DOM-2	7.74	4.59	0.2	0.03	3.73
	DOM-3	8.93	7.09	0.68	0.03	4.39
Summer	DOM-1	8.29	6.9	0.27	6.9	9.48
	DOM-2	7.84	7.64	0.20	7.64	8.93
	DOM-3	7.75	8.16	0.44	8.16	10.33
Autumn	DOM-1	8.28	4.75	0.21	4.75	11.52
	DOM-2	7.08	4.6	0.02	4.6	8.58
	DOM-3	8.18	5.57	0.05	5.57	12.72
Winter	DOM-1	7.81	7.11	0.07	0.04	6.06
	DOM-2	7.01	5.13	0.08	0.38	4.25
	DOM-3	7.66	5.29	0.05	0.04	5.83

Table S6. The main information on photodegradation products of target pollutants.

Products No.	m/z $[M+H]^+$	Retention time (min)	Molecular formula	Structural formula
LFX	362.1511	4.528	$C_{18}H_{20}FN_3O_4$	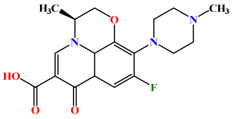
L1	378.1460	12.8	$C_{16}H_{21}FN_3O_5$	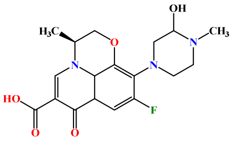
L2	336.1354	11.5	$C_{16}H_{21}FN_3O_4^+$	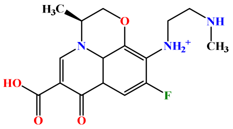
L3	279.0776	15	$C_{13}H_{10}FN_2O_3$	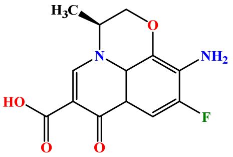
L4	234.08	10.7	$C_{12}H_{11}FN_2O_2$	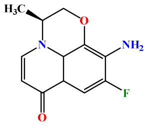
L5	349.14	11.86	$C_{17}H_{18}FN_3O_4$	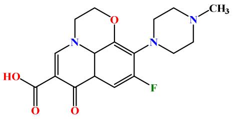
L6	191	12.25	$C_{11}H_{10}FNO$	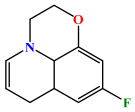
L7	380.16	1.4	$C_{18}H_{23}FN_3O_5^+$	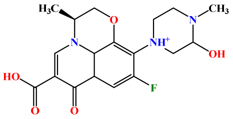
L8	366.15	15.2	$C_{17}H_{21}FN_3O_5^+$	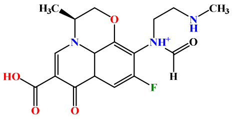
SMZ	254.0594	8.11	$C_{10}H_{11}N_3O_3S$	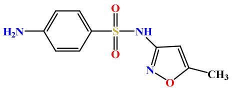
S1	268.03	7.4	$C_{10}H_9N_3O_4S$	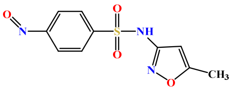

S2	172.99	0.86	$C_6H_5NO_3S$	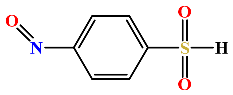
S3	107.06	0.91	C_6H_7NO	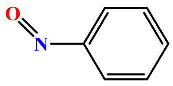
S4	123.03	0.92	$C_6H_5NO_2$	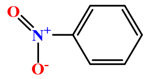
S5	269.0536	0.92	$C_{10}H_{11}N_3O_4S$	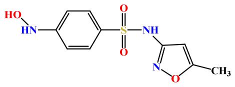
S6	264.03	8.26	$C_{10}H_{10}N_3O_5S^+$	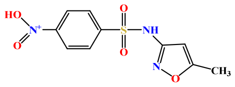
S7	270.05	14.3	$C_{10}H_{11}N_3O_4S$	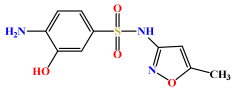
S8	189.0157	2.61	$C_6H_7N_4OS$	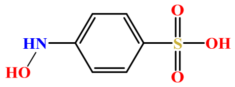
IBP	206.13	19.82	$C_{18}H_{18}O_2$	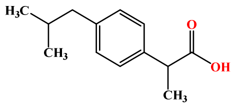
P1	161.13	16.01	$C_{12}H_{17}^+$	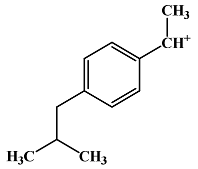
P2	195.15	5.61	$C_{12}H_{19}O_2^+$	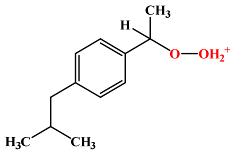
P3	176.12	21.03	$C_{12}H_{16}O$	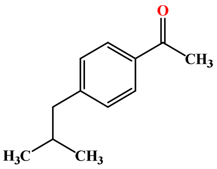
P4	178.14	20.28	$C_{12}H_{18}O$	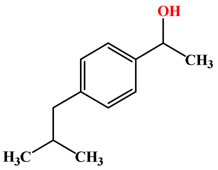
P5	166.06	19.14	$C_9H_{10}O$	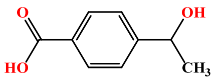
P6	164.05	23.59	$C_9H_8O_3$	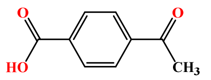

P7	178.10	20.44	$C_{11}H_{14}O_2$	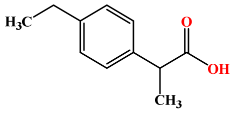
P8	134.11	24.56	$C_{10}H_{14}$	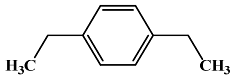
P9	164.08	2.19	$C_{10}H_{12}O_2$	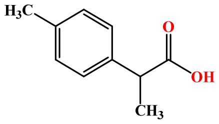
P10	162.14	18.55	$C_{12}H_{18}$	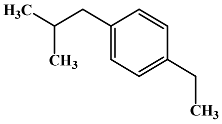
P11	134.11	14.62	$C_{10}H_{14}$	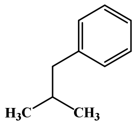

Table S7. Possible photodegradation by-products and their acute toxicity.

Number	m/z	Molecular Formula	Possible Structures	Organism	Predicted (mg/L)
L1	378.1460	$C_{16}H_{21}FN_3O_5$	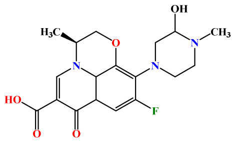	LC_{50}	2.81e + 005
L2	336.1354	$C_{16}H_{21}FN_3O_4$ +	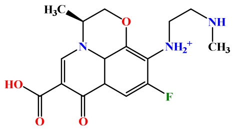	null	null
L3	279.0776	$C_{13}H_{10}FN_2O_3$	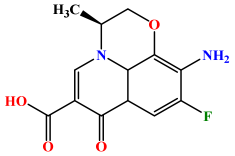	LC_{50}	5.24e + 005
L4	234.08	$C_{12}H_{11}FN_2O_2$	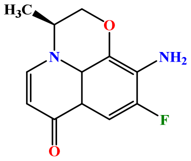	LC_{50}	232.474
L5	349.14	$C_{17}H_{18}FN_3O_4$	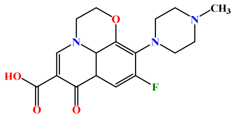	LC_{50}	69051.555

L6	191	$C_{11}H_{10}FNO$	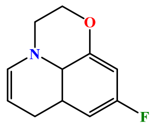	LC_{50}	7.364
L7	380.16	$C_{18}H_{23}FN_3O_5^+$	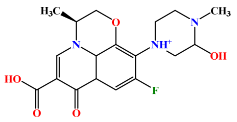	null	null
L8	366.15	$C_{17}H_{21}FN_3O_5^+$	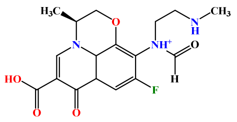	null	null
S1	268.03	$C_{10}H_9N_3O_4S$	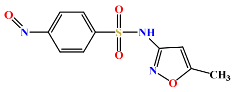	LC_{50}	56.831
S2	172.99	$C_6H_5NO_3S$	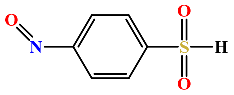	LC_{50}	3946.822
S3	107.06	C_6H_7NO	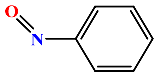	LC_{50}	60.202
S4	123.03	$C_6H_5NO_2$	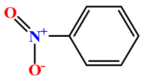	null	null
S5	269.0536	$C_{10}H_{11}N_3O_4S$	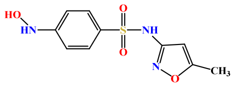	LC_{50}	194.836
S6	264.03	$C_{10}H_{10}N_3O_5S^+$	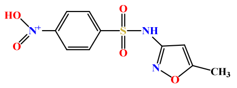	null	null
S7	270.05	$C_{10}H_{11}N_3O_4S$	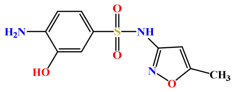	LC_{50}	6.369
S8	189.0157	$C_6H_7N_4OS$	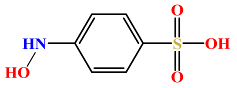	LC_{50}	1.9e + 006
P1	161.13	$C_{12}H_{17}^+$	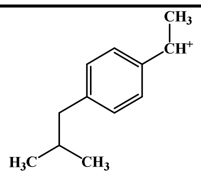	null	null
P2	195.1492	$C_{12}H_{19}O_2^+$	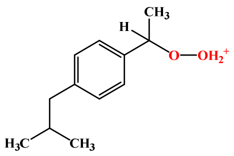	null	null

P3	176.12	$C_{12}H_{16}O$	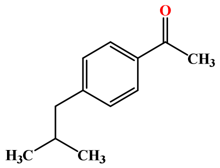	LC_{50}	3.650
P4	178.14	$C_{12}H_{18}O$	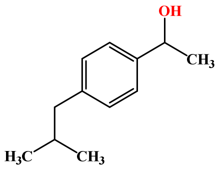	LC_{50}	0.548
P5	166.06	$C_9H_{10}O$	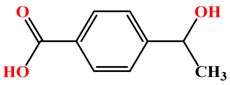	LC_{50}	384.284
P6	164.05	$C_9H_8O_3$	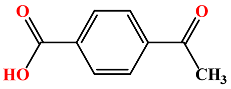	LC_{50}	1645.733
P7	178.10	$C_{11}H_{14}O_2$	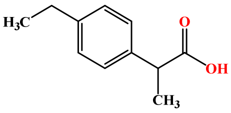	LC_{50}	340.937
P8	134.11	$C_{10}H_{14}$	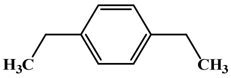	LC_{50}	1.196
P9	164.08	$C_{10}H_{12}O_2$	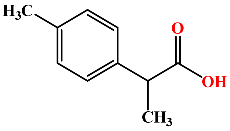	LC_{50}	141.124
P10	162.14	$C_{12}H_{18}$	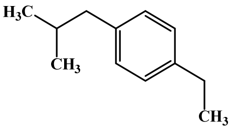	LC_{50}	0.262
P11	134.11	$C_{10}H_{14}$	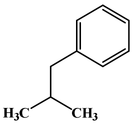	LC_{50}	1.525

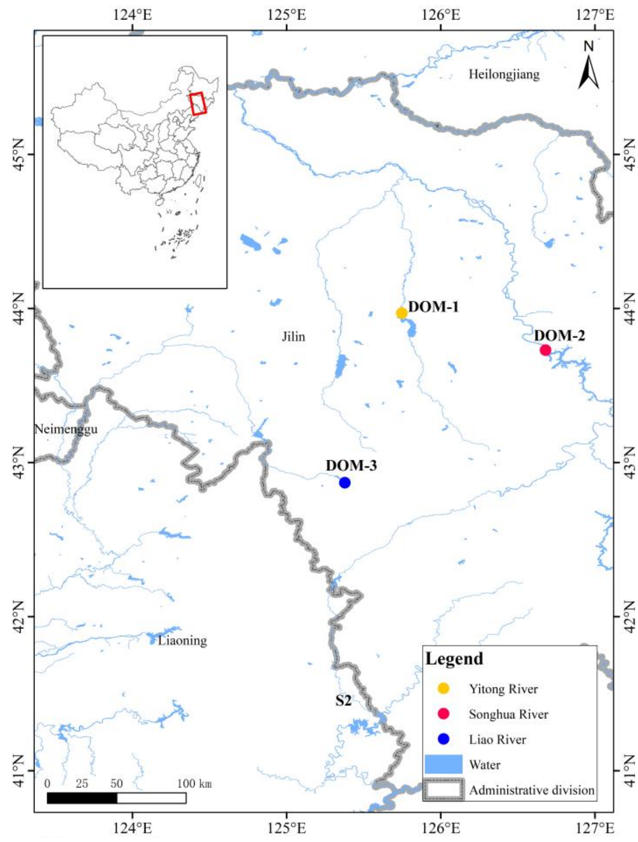

Figure S1. Map of sampling sites.

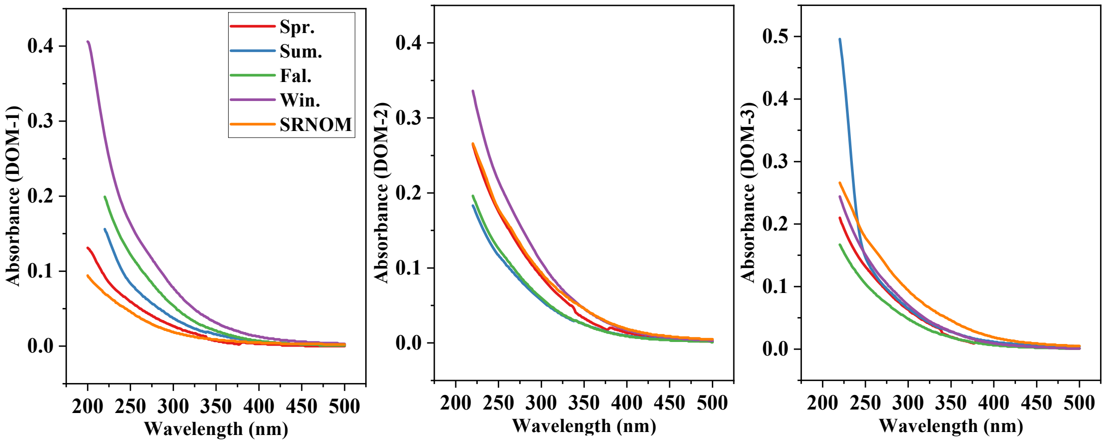

Figure S2. UV-vis absorbance of DOM in different seasons and SRNOM, [DOM] = 5 mgC L^{-1}.

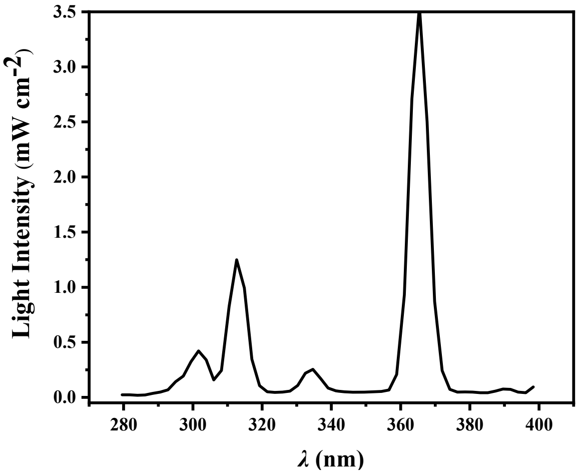

Figure S3. Emission spectrum of the 500 W Hg lamp at λ = 280 - 400 nm.

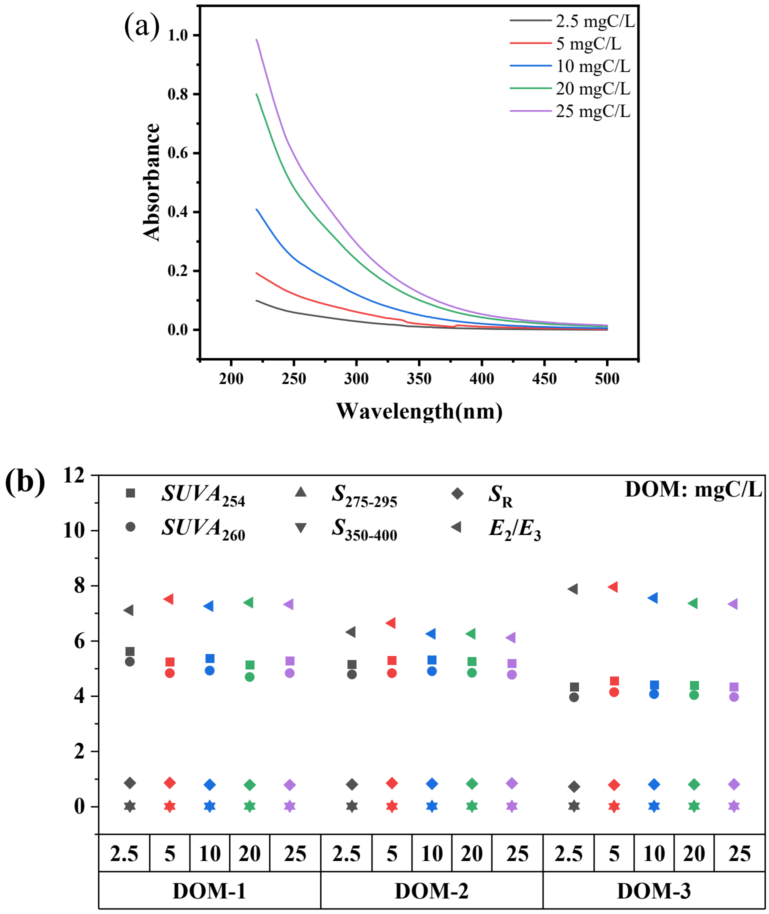

Figure S4. UV absorption spectra (a) and spectral parameters (b) for different concentrations of DOM.
